# Factors affecting viral suppression or rebound in people living with HIV and receiving antiretroviral therapy in Ghana

**DOI:** 10.3389/fpubh.2025.1508793

**Published:** 2025-03-19

**Authors:** Anthony T. Boateng, James O. Aboagye, Helena Lamptey, Christopher Z.-Y. Abana, Araba Abaidoo-Myles, Darius N. K. Quansah, Seth Agyemang, Yaw Awuku-Larbi, Gloria Ansa, Joseph Oliver-Commey, Vincent Ganu, George B. Kyei, Peter Puplampu, Evelyn Y. Bonney

**Affiliations:** ^1^Department of Virology, Noguchi Memorial Institute for Medical Research, College of Health Sciences, University of Ghana, Accra, Ghana; ^2^West African Centre for Cell Biology of Infectious Pathogens, Department of Biochemistry, Cell and Molecular Biology, College of Basic and Applied Sciences, University of Ghana, Accra, Ghana; ^3^Medical and Scientific Research Center, University of Ghana Medical Center, Accra, Ghana; ^4^Department of Immunology, Noguchi Memorial Institute for Medical Research, College of Health Sciences, University of Ghana, Accra, Ghana; ^5^Central Laboratory, Immunology Department, Korle-Bu Teaching Hospital, Accra, Ghana; ^6^Public Health Unit, University of Ghana Hospital, Accra, Ghana; ^7^LEKMA Hospital, Accra, Ghana; ^8^Department of Medicine, University of Ghana School of Medicine, College of Health Sciences, University of Ghana, Accra, Ghana; ^9^Departments of Medicine and Molecular Microbiology, Washington University School of Medicine, St Louis, MO, United States

**Keywords:** viral rebound, viral suppression, viral load, ART, HIV

## Abstract

**Introduction:**

Regular viral load (VL) testing for people living with HIV (PLWH) is key to attaining the Joint United Nations Program on HIV/AIDS (UNAIDS) Fast-Track 95–95-95 target to end the HIV epidemic by 2030. However, VL testing remains sporadic in routine HIV care in the majority of resource-limited settings, including Ghana, except when provided through research initiatives. In this study, we measured VL among PLWH in Ghana at regular intervals and investigated factors affecting viral suppression (VS) and rebound.

**Methods:**

We analyzed data from a hospital-based cohort enrolled in our HIV cure research. Participants were recruited from three hospitals in the Greater Accra region of Ghana. Demographic characteristics were obtained from participants’ folders, while CD4^+^ T cell counts and VLs were measured from blood samples collected at baseline, 6 months, and 18 months.

**Results:**

The study participants were predominantly women (68%) with a median age of 45 years (IQR: 21–76 years). A total of 52% of participants had been on antiretroviral therapy (ART) for more than 6 years, and 74% were following dolutegravir-based regimens. At baseline, 74% of participants had a VL of <50 copies/mL, which increased to 88% at 18 months, with 80% having a CD4^+^ T cell count of >350 cells/μl. Age group [<40 vs. > 40 years] (OR 2.35, 95% CI; 1.21–4.58, *p* = 0.012), CD4^+^ T cell count [>350 vs. < 350 cells/μl] (OR 4.35, 95% CI; 2.32–8.18, *p* < 0.001), and ART regimen [NVP based vs. DTG based] (OR 7.00, 95% CI; 1.15–42.57, *p* = 0.034) were associated with VS of <50 copies/mL. The overall viral rebound rate was estimated at 13.61 per 1,000 person-months (95% CI 10.52–17.74), with decreasing rates over time. Lower educational levels (up to Junior High School) were significantly associated with viral rebound (*p* = 0.011).

**Conclusion:**

A key feature of our study was measuring VL at three time points over 2 years, which may explain the high VS levels observed. Viral rebound was linked to low education levels, highlighting the need for targeted education for PLWH with junior high school (JHS) education or less. Regular VL monitoring and the implementation of measures to prevent viral rebound, particularly among PLWH with lower education levels, will help Ghana move closer to attaining the third “95” of the UNAIDS 95–95-95 target by 2030.

## Introduction

Human immunodeficiency virus (HIV) infection continues to be a global pandemic, with over 38 million people living with the virus; the majority of the infected people are in sub-Saharan Africa (SSA) (1). The widespread use of antiretroviral therapy (ART), which can suppress the virus to undetectable levels, has transformed HIV infection into a manageable chronic disease requiring daily medication (2–4).

The Joint United Nations Program on HIV/AIDS (UNAIDS) has set ambitious targets to control the HIV epidemic by 2030, aiming for 95% of people living with HIV (PLWH) to know their status, 95% of those diagnosed to be on ART (antiretroviral therapy), and 95% of those on ART to achieve viral suppression (VS). Achieving VS is key to reducing transmission and disease progression, making it a critical goal for everyone receiving treatment (5–11).

The factors likely to affect VS include the type of antiretroviral (ARV) regimen, patient adherence, and the frequency of VL measurements to facilitate timely regimen adjustments (12). The sporadic nature of viral load testing in SSA and other resource-limited settings (RLS), coupled with less potent first-generation ARVs that have burdensome side effects hindering adherence, promotes drug resistance and viral non-suppression. Therefore, the World Health Organization (WHO) recommended dolutegravir (DTG), a second-generation integrase strand transfer inhibitor (INSTI), in 2018 to be used in combination with two NRTIs as a first-line regimen for SSA and other RLS to improve VS. Following this recommendation, Ghana transitioned from a first-line regimen based on non-nucleotide reverse transcriptase inhibitors (NNRTIs) such as Nevirapine (NVP) or Efavirenz (EFV), combined with two nucleos(t)ide reverse transcriptase inhibitors (NRTIs) including Lamivudine (3TC), Zidovudine (AZT), or Tenofovir disoproxil fumarate (TDF), to a DTG-based regimen (13–15). This switch aimed to improve adherence, reduce drug resistance, and improve VS rates in SSA (16, 17).

Therefore, measuring VS is imperative to ascertain the effectiveness of the new drug regimens and determine the factors that affect it.

Measuring VL is the primary method for assessing the effectiveness of ART, as it provides critical insights into VS (18, 19). However, achieving VS in PLWH is not a stable or permanent condition. Some individuals may lose their VS status over time and experience viral rebounds (20). Viral rebound is known to be associated with active tuberculosis, ART adherence, duration of prior suppression, education levels, and the type of ARVs used (12, 20, 21). Additionally, socioeconomic factors such as disparities in education and income—manifesting as health literacy and financial barriers—may improve non-ART adherence, potentially impacting VS and rebound rates. Viral rebounds can undermine the gains made by ART by increasing treatment failure and ART resistance, thus facilitating HIV transmission (21). The WHO recommends initiating treatment and monitoring response by performing the first VL measurement 6 months after treatment initiation, followed by yearly intervals if suppression is achieved (22). However, these guidelines are not consistently followed in SSA, hindering the early detection of virologic failure. Previous studies have shown an increase in HIV drug resistance in Africa due to irregular monitoring of VL and the lack of drug resistance testing before changing drug regimens (23, 24).

According to the Ghana AIDS Commission, Ghana’s VS rate is 68%, which is significantly below UNAIDS target of 95%. To assess progress toward the third “95” of the UNAIDS 95–95-95 target and identify necessary interventions to maintain VS, it is crucial to evaluate trends in VS, instances of viral rebound, and the factors influencing these two outcomes.

In this study, we report the levels of VS, viral rebound rates, and factors associated with VS and rebound among PLWH over 18 months.

## Materials and methods

### Study design

This is a secondary data analysis using data from a hospital-based cohort study aimed at identifying virologically suppressed individuals for HIV cure research. The cohort study was conducted at three hospitals in the Greater Accra region of Ghana: Korle-Bu Teaching Hospital, LEKMA Hospital, Teshie, and University of Ghana Hospital, Legon. Demographic characteristics were obtained from participants’ records, and blood samples were collected from patients to measure CD4^+^ T cell counts and VLs at baseline, 6 months, and 18 months.

### Study population and sampling

Between 2019 and 2021, the parent study enrolled 390 PLWH on ART from three hospitals in the Greater Accra region of Ghana. A total of 250 participants were recruited from Korle-Bu Teaching Hospital (KBTH), and 70 each were recruited from LEKMA Hospital and the University of Ghana Hospital, Legon. Details of the study hospitals have been previously published (25). Participants were enrolled at baseline and followed up at 6 and 18 months over a 2-year period.

At each sampling time point, 10 mL of venous blood was collected into EDTA tubes from participants and transported in cool boxes to the Noguchi Memorial Institute for Medical Research (NMIMR). Aliquots of the whole blood were used to estimate CD4^+^ T cell count, while the remaining blood was processed into plasma and peripheral blood mononuclear cells (PBMCs) using the sucrose-gradient centrifugation method. Plasma was stored at -20°C and PBMCs at −80°C in a freezing medium (FBS with 10% DMSO) until further laboratory analysis. The participants’ demographics and treatment history, including age, sex, educational status, income level, ART regimen, and occupation, were obtained from hospital records.

### Inclusion/exclusion criteria

All consenting ART-experienced PLWH aged 18 years and older were included in the parent study. All PLWH under 18 years of age and ART naïve were excluded. For this study, PLWH from the parent study with VL results at all three follow-up points were analyzed to identify factors associated with VS and rebound.

### Viral load determination

VL was measured from the plasma using the COBAS^®^ AmpliPrep/COBAS^®^ TaqMan^®^ following the manufacturer’s instructions (Roche Diagnostic Systems, Branchburg, NJ, United States). For quality control, a negative control, HIV-1 Low Positive Control, and HIV-1 High Positive Control were included in each test batch. This assay can quantify HIV-1 RNA with a lower detection limit of 20 copies/mL and an upper limit of 10,000,000 copies/mL. One copy of HIV-1 RNA is equivalent to 1.7 ± 0.1 International Units (IU) based on the WHO 1st International Standard for HIV-1 RNA for Nucleic Acid-Based Techniques (NAT; NIBSC 97/656).

### CD4^+^ T and CD8^+^ T cell measurement

Whole blood samples were analyzed for CD4^+^ T and CD8 ^+^T cell counts using the FACS Count system (Becton Dickinson Biosciences, United States), according to the manufacturer’s instructions. Briefly, 50 μL of blood was added to CD4 and CD8 reagent tubes and incubated for 1 h at room temperature in the dark, in accordance with the manufacturer’s protocol. A fixative solution (50 μL) was added and analyzed on the FACS Count machine to enumerate the absolute CD4 ^+^ and CD8^+^ T cells per microliter of blood. The equipment was also calibrated to validate the results, and reagent controls were run alongside each batch of samples.

### Statistical analysis

Participants’ characteristics were analyzed descriptively using frequency distribution tables and graphical presentations. Data capture, cleaning, coding, and analysis were conducted using.

Microsoft Excel spreadsheets (Excel 2016) and STATA 16. VS was defined as the proportion of individuals with HIV VL <50 copies/mL and [<1,000 copies/mL for the WHO criteria for resource-limited settings (RLS)]. Frequencies and percentages were used to describe categorical outcomes, while medians and interquartile ranges were used to summarize continuous variables. Odds ratios in the multivariable model were calculated to examine factors associated with VS using a generalized estimating equation with a binomial distribution and logit link function to account for within-subject correlation. The HIV rebound rate was defined as the number of participants who were unsuppressed following VS with results <50 copies/mL over the study period from enrollment to the second follow-up. The viral rebound was calculated as a rate per 1,000 person-months using survival analysis. Confidence intervals were reported, and *p*-values of <0.05 were considered statistically significant.

## Results

### Participants’ characteristics

A total of 390 PLWH in Ghana were recruited from three hospitals in Accra for this study. In total, 265, representing 68% of the participants, were women, while the remaining 32% were men (Table 1). The median age of the participants was 45 years (IQR 21–76). The majority had only a minimum level of formal education; only 29% of them had completed high school or pursued further education. Participants had a low economic status, with 71% earning less than $100 USD per month. The median duration since the HIV diagnosis was 7 years (IQR 0–30), and 59% of participants had been on antiretroviral treatment for more than 6 years (Table 1). The estimated CD4^+^ T cell counts for 80% of the participants were > 350 cells/μL. Of the 390 participants, VL results were available for 361 at baseline, 325 at 6 months, and 321 at 18 months (Table 1).

**Table 1:** Demographic and infection history of study participants.

Variables	Number (N)	Percentage
Age group (years) *N* = 390		
20–40	111	28.46
41–50	165	42.31
>50	114	29.23
Median (IQR)	45 (21–76)	
Sex *N* = 390		
Female	265	67.95
Male	125	32.05
Education level *N* = 387		
≤Primary	119	30.75
JHS/Middle School	154	39.79
≥Secondary School	114	29.46
Monthly income (GHC) *N* = 383		
≤200	147	38.38
201–500	131	34.20
>500	105	27.42
Years diagnosed *N* = 380		
<5	128	33.68
5–9	115	30.26
≥10	137	36.05
Median (IQR)	7 (0–30) years	
Treatment duration *N* = 382		
≤6	183	47.91
>6	199	52.09
CD4 Count *N* = 390		
<350	78	20.00
≥350	312	80.00
Median (IQR)	594 (2–2053)	
ART regimen *N* = 324		
DTG-based	241	74.38
EFV-based	67	20.68
NVP-based	16	4.94
Number of participants with VL results per timepoint		
Baseline	361/390	92.56
6 months	325/390	83.33
18 months	321/390	82.31

### VS per sampling time points

**Figure 1:**
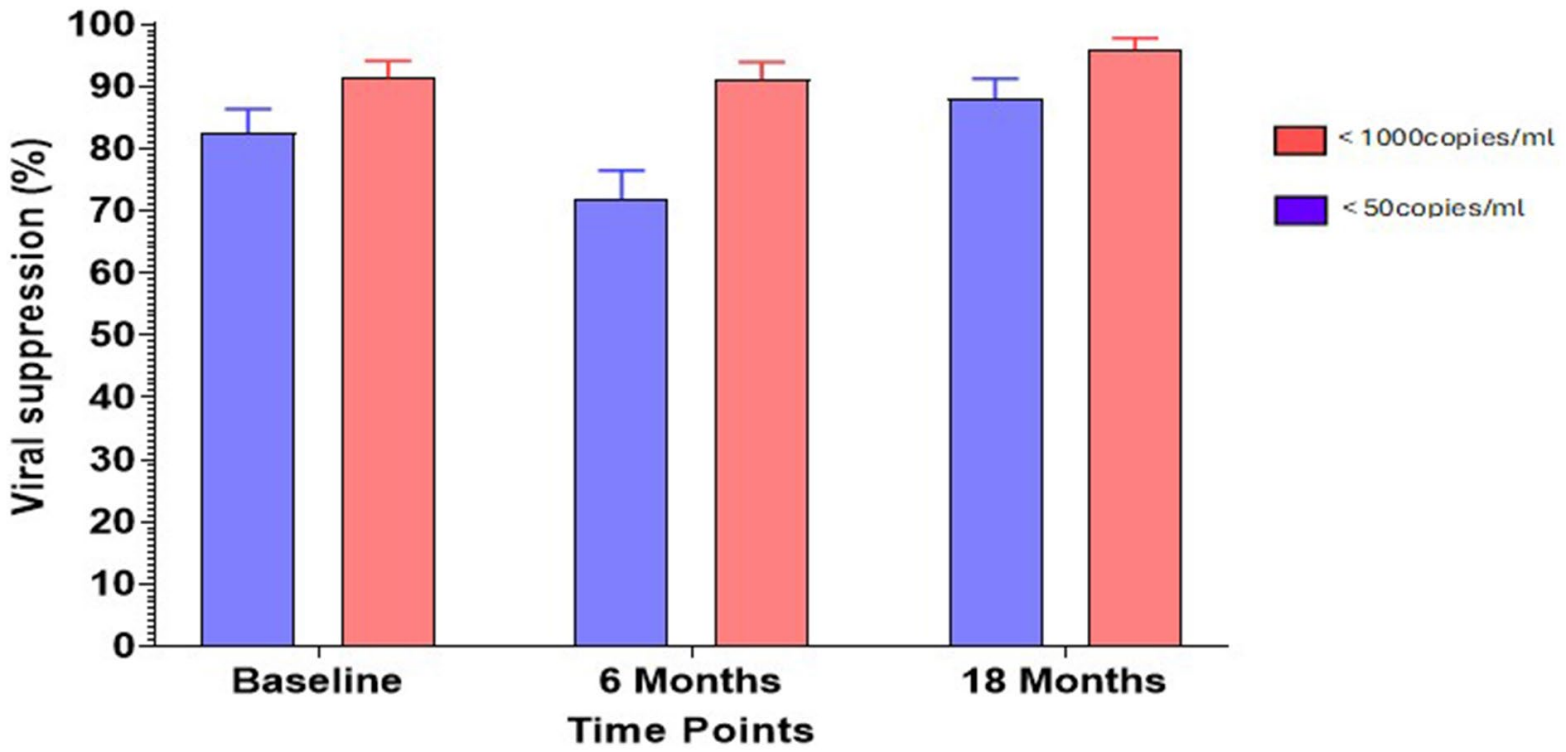
Percentage viral suppression at sampling timepoints. Viral suppression was based on the thresholds set at <50 copies/ml (purple bars) and <1,000 copies/ml (pink bars).

At recruitment, 268 out of 390 participants, representing 74%, had VLs less than 50 copies/mL, and 91% had VLs less than 1,000 copies/mL. At 6 months, out of 325 participants, 73% of the participants had VL less than 50 copies/mL, and 91% of the participants had VL less than 1,000 copies/mL. At 18 months, 88% of 321 participants had VLs less than 50 copies/mL, and 96% of the participants had VLs less than 1,000 copies/mL (Figure 1).

### VS trends of compliant participants

In total, 275 participants had their VLs measured at all three time points in the study.

**Figure 2:**
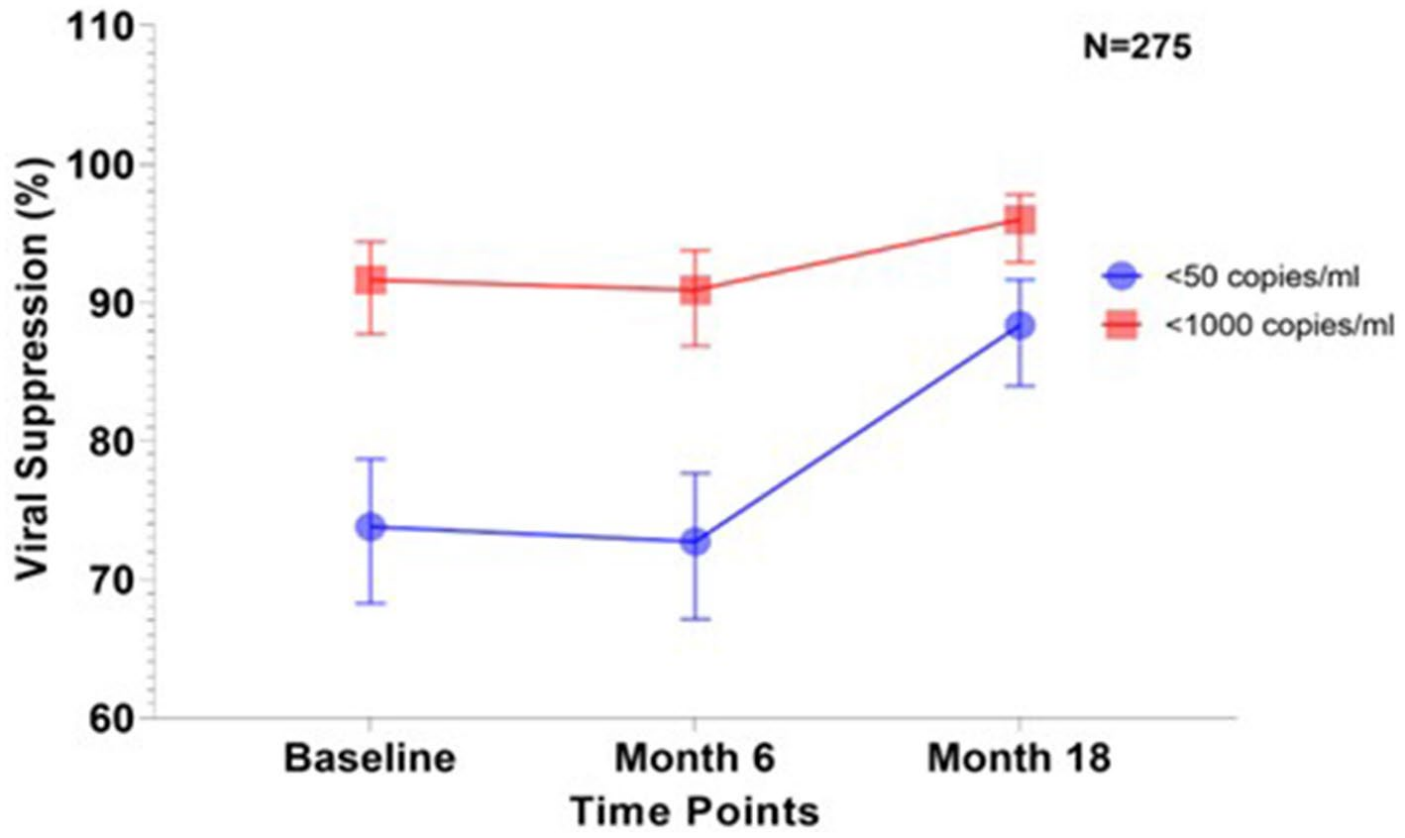
Viral suppression among patients whose viral loads were successfully measured at all the three sampling timepoints (*N* = 275). The graph shows the trend in the proportion of virally suppressed individuals and their confidence interval in percentages over three-time points; baseline, 6 and 18 months according to the two thresholds (<50 copies/ml and <1,000 copies/ml).

Among these, VS <50 copies/mL was observed in 74, 73, and 88% at baseline, 6 months, and 18 months (Figure 2). At all three time points, VS levels exceeded 90% when using the WHO threshold of <1,000 copies/mL (Figure 2).

### Factors associated with VS

**Figure 3:**
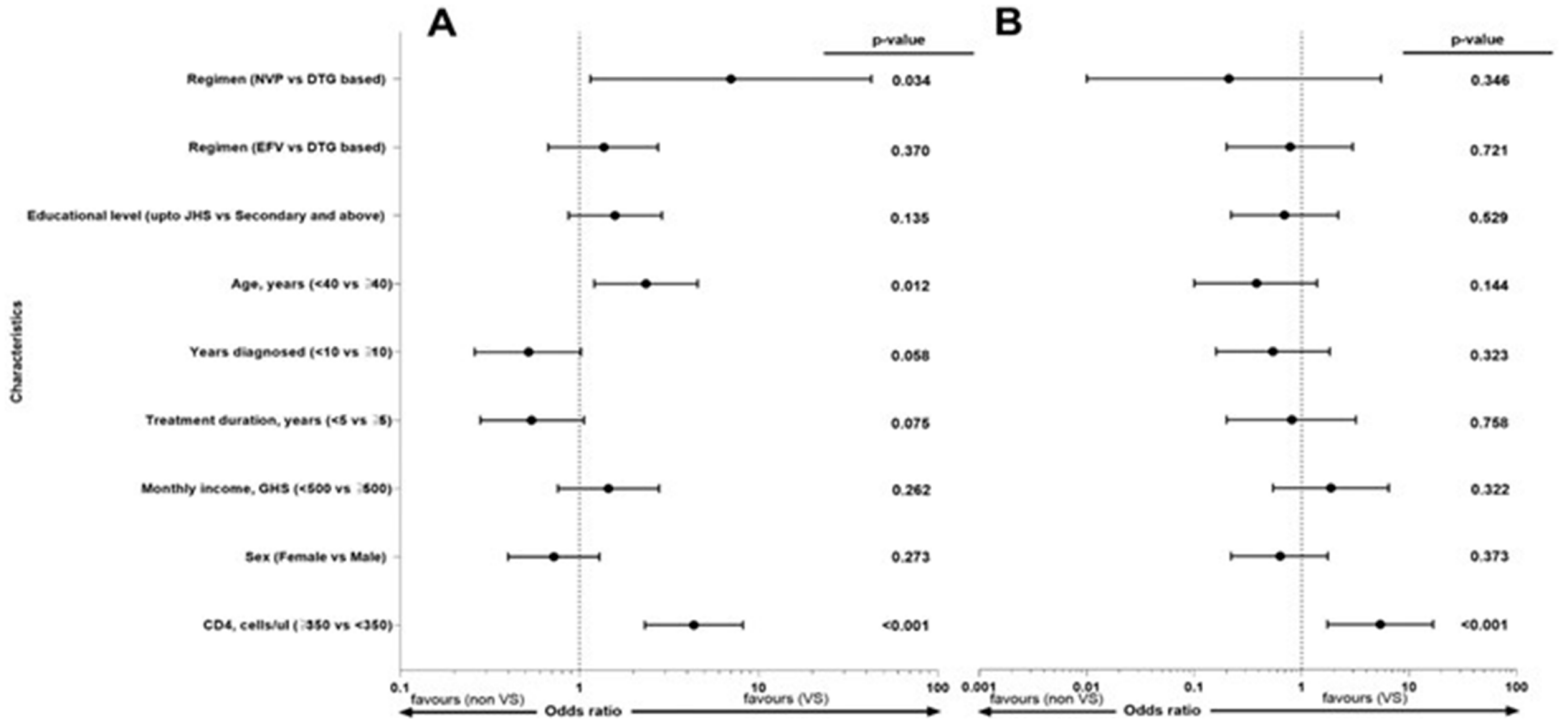
Factors associated with viral suppression for thresholds **(A)** <50 copies/ml and **(B)** from 51 to 1,000 copies/ml. The forest plot shows the factors associated with HIV suppression (<50 copies/ml, *N* = 275: 51–1,000 copies/ml, *N* = 179). The dashed lines represent the reference point = 1, the black dot represents the point odds ratio estimate, and the whiskers represents the confidence intervals. *p*-values <0.05 are considered significant. VS, viral suppression.

We examined the factors associated with VLS at the thresholds <50 copies/mL and from 51 to 1,000 copies/mL. Age group (OR 2.35, 95% CI; 1.21–4.58, *p*-value = 0.012), CD4^+^ T cell count (OR 4.35, 95% CI; 2.32–8.18, *p*-value<0.001), and ART regimen [NVP based vs. DTG based] (OR 7.0, 95% CI; 1.15–42.57, *p*-value = 0.034) were associated with VLS with threshold of <50 copies/mL, while CD4^+^ T cell count (OR 5.39, 95% CI; 1.73–16.73, *p*-value<0.001) was associated an VL of of 51 to 1,000 copies/mL (Figure 3).

### Viral rebound rates

**Figure 4:**
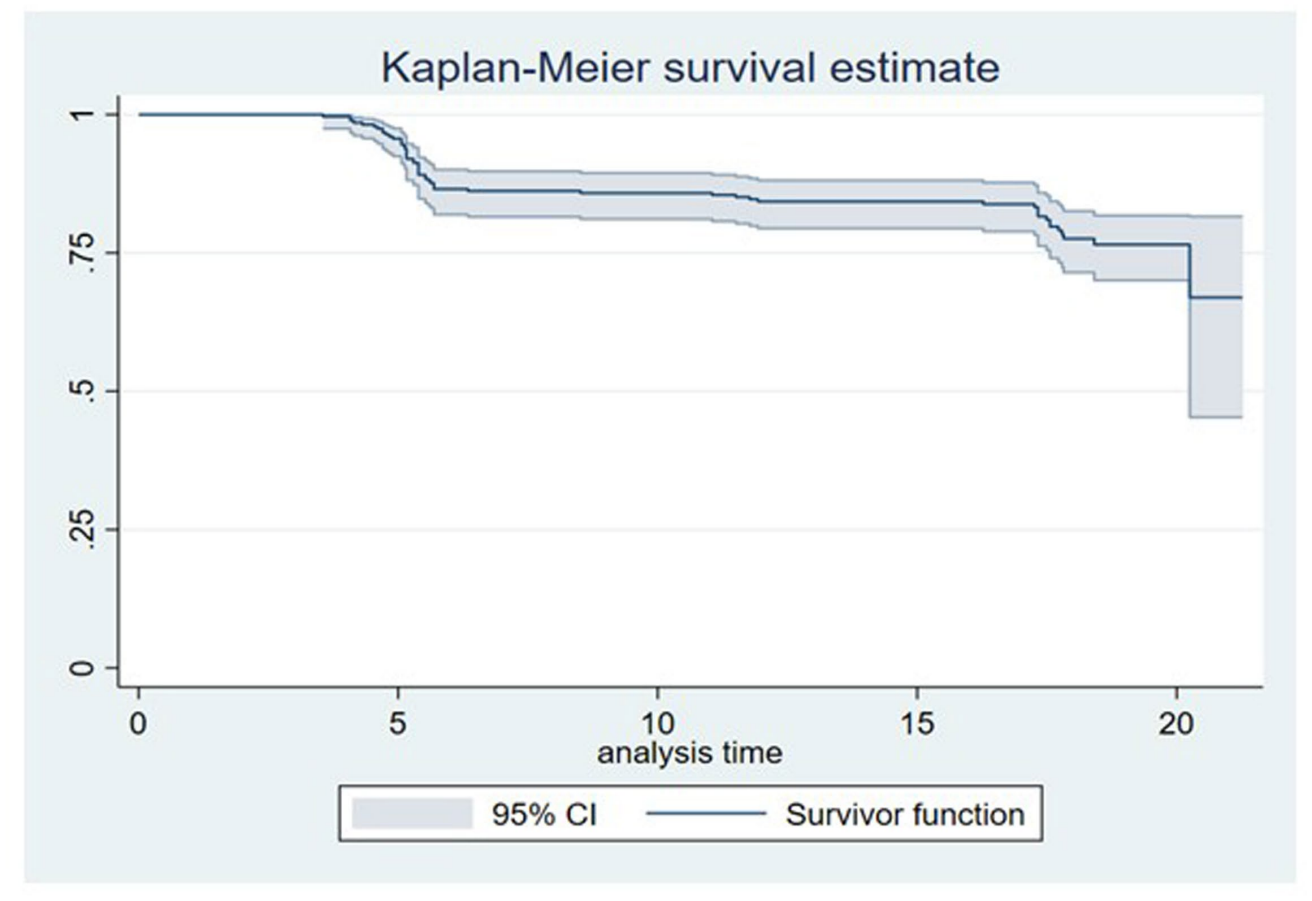
Kaplan Meier curve showing the distribution of rebound cases over the follow-up period.

We followed 275 participants with VL results across the follow-up points, totaling 4,120 person-months (Table 2). During the follow-up period, 56 participants experienced HIV rebound. At 6, 12, and 18 months, 43, 11, and 2 participants, respectively, experienced viral rebound (Figure 4). The overall viral rebound rate was estimated at 13.61 per 1,000 person-months (95% CI 10.52–17.74; Table 2).

**Table 2:** Showing the distribution of rebound rates by covariates.

Characteristic	Events	Number	Time at risk (Person months)	Crude IR (95%CI)	*p-*value
Overall	56	275	4120	13.59 (10.46–17.66)	
Sex					0.926
Male	20	99	1490	13.42 (8.66–20.80)	
Female	36	176	2629	13.69 (9.88–18.98)	
Age (years)					0.904
<40	14	70	1034	13.54 (8.02–22.86)	
≥40	42	205	3086	13.61 (10.06–18.42)	
Educational level					0.011
Up to Junior High School	47	190	2839	16.55 (12.44–22.03)	
Senior Secondary and above	8	83	1263	6.33 (3.17–12.66)	
Years diagnosed with HIV					0.123
<10	30	796	2617	11.46 (8.01–16.39)	
≥10	24	92	1397	17.18 (11.51–25.63)	
Years on ART					0.077
<5	15	107	1586	9.46 (5.7–15.69)	
≥5	40	163	2470	16.19 (11.9–22.08)	
Monthly income (GHS)					0.064
<500	45	192	2820	15.96 (11.91–21.37)	
≥500	11	80	1254	8.77 (4.86–15.84)	
ART Regimen					0.851
DTG based	34	178	2668	12.74 (9.1–17.83)	
EFV based	7	42	647	10.81 (5.16–22.69)	
NVP based	2	10	152	13.14 (3.29–52.56)	
CD4, (Copies/mL)					0.373
<350	5	37	263	19.0 (7.91–45.67)	
≥350	36	211	1315	27.37 (19.74–37.94)	

### Factors associated with viral rebound

The HIV rebound rate was significantly higher among participants with educational levels up to junior high school (16.55 per 1,000 person-months) compared to those with educational levels from senior secondary school and above (6.33 per 1,000 person-months), *p* = 0.011. However, the years on ART and monthly income were marginally significant (Table 2).

## Discussion

In this study, we determined VS/rebound in participants on ART in Ghana who enrolled in our HIV cure studies and received regular VL measurements over a 2-year period. Prior to our study, regular VL measurements were not routinely conducted for participants.

Women constituted 68% of our study population. This aligns with national HIV statistics in Ghana, where women account for two-thirds of PLWH (26). The age group of 41 to 50 years represented 42% of the study population, indicating that Ghana has an aging HIV population that requires targeted management to ensure they remain healthy and can contribute to national development. Over 62% had been diagnosed with HIV at least 5 years before sampling, and 27% of them had been aware of their status for more than 10 years before the study. In total, 59% had been on ART for over 6 years before sampling. Some participants did not start ART at the time of diagnosis, probably due to their CD4^+^ T cell counts and the policy of initiating ART at a specified CD4^+^ T cell threshold.

Achieving VS is the optimal goal of antiretroviral therapy, with VL measurements being the best tool for assessment (27). Various factors determine whether patients on therapy will attain VS, including the time from infection to initiation of ART, the type of antiretrovirals prescribed, the duration of treatment, adherence to the treatment regimen, and the patients’ age and sex. While a longer duration of treatment is generally a positive indicator for achieving VS, remaining on an ineffective regimen for too long could negate the benefits of ART. This study considers educational level and income as key determinants of VS, given their critical role in an individual’s ability to access healthcare and adhere to prescribed treatment.

Higher levels of education enhance individuals’ ability to make informed healthcare decisions and understand medical instructions. Similarly, income levels can directly impact one’s ability to afford healthcare services and transportation to medical facilities.

In resource-limited settings such as Ghana, VL measurements are not routinely conducted for people on ART. However, the treatment guidelines recommend VL measurements at 6-month intervals until suppression and once per year thereafter (28). This is due to limited resources such as equipment, ineffective sample transport systems, a lack of skilled personnel at the peripheral laboratories to analyze the samples, and non-adherence to ART guidelines by healthcare workers.

In this study, we measured the VLs for all participants sampled at the three-time points.

The unavailability of VL results for some participants (Table 1) was due to either invalid test results or participant loss to follow-up.

We found that 74% of participants in our cohort had a VL of <50 copies/mL, which is similar to the 76% VS observed by Opoku et al. in a study conducted at Komfo Anokye Teaching Hospital in Kumasi, Ghana (20). This presents a positive outlook for Ghana’s ART program and may suggest that the drugs are generally effective. Among participants with VL ≥ 50 copies/mL, 62 (17%) had VL of less than 1,000 copies/mL; thus, a total of 91% of participants met the World Health Organization’s criteria for VS in RLS. Antiretrovirals used in Ghana were mostly first-generation ARVs until the introduction of DTG into the first-line regimen in 2019 (29). The treatment regimens in Ghana primarily consisted of reverse transcriptase inhibitors, with protease inhibitors introduced during the second-line regimen when the first-line regimen failed (30). The level of VS found in our study corroborates findings from previous studies by Ali et al., who reported 72% VS (<1,000 copies/mL) from Ethiopia; Wakooko et al., with 87.5% VS (<1,000 copies/mL) from Eastern Uganda; Mogosetsi et al., who reported 91% VS (<1,000 copies/mL) in South Africa; Abdullahi et al., who reported 91% VS (<1,000 copies/mL) from northwestern Nigeria; and Djiyou et al., reporting 88.2% VS (<1,000 copies/mL) from Cameroon (27, 31–34). Other African countries have reported lower levels: 59% in Kenya (12) and 56.2% among pregnant women in South Africa (35).

VS levels at the three sampling time points showed an increasing trend, which could be attributed to the introduction of DTG (Figure 1). The slight dip observed in Follow-up 1 (2020) could be due to the impact of the COVID-19 pandemic, which drastically reduced access to healthcare (36). Overall, the high levels of suppression in our participants over the study period could be due to the availability of VL testing for each participant in our cohort, which guided monitoring for improved virologic outcomes.

Age, ART regimen, and CD4^+^ T cell count were associated with VS (VL < 50 copies/mL), while only CD4^+^ T cell count was associated with VLs between 51 and 1,000 copies/mL. Participants older than 40 years were more likely to be virally suppressed compared to those younger than 40 years. This contrasts with findings from a study in Canada, where adults under 29 years experienced a lower prevalence of VS (37). This may indicate that Ghana’s older population is more receptive to and compliant with their treatment than other populations. A nevirapine-based regimen was more likely to be associated with VS than dolutegravir. This observation could be attributed to factors such as adherence differences, sampling time, and, more importantly, our cohort composition bias. Dolutegravir (DTG) was introduced just before the study, and at baseline, a higher proportion of ART-experienced participants who were doing well were on nevirapine-based regimens, leaving only the newly initiated patients or those with prior virologic failure and higher VLs on DTG-based regimens. However, at month 18, a larger proportion of participants were on a DTG-based regimen and had their VLs below 50 copies/mL, demonstrating the superior efficacy of DTG, which aligns with global evidence.

Achieving VS has many benefits for PLWH because it drastically reduces HIV transmission risk (undetectable equals untransmissible). This is particularly beneficial to discordant couples and babies born to women with HIV (38, 39).

Viral rebounds pose a significant risk to HIV morbidity and mortality. In our study, viral rebound occurred in 20.4% of individuals, with the rebound rate estimated at 13.61 per 1,000 person-years (95% CI 10.52–17.74). This is comparable to the findings by Opoku et al., who reported a 21% viral rebound rate among PLWH in Ghana (20). This finding in our cohort confirms that viral rebounds occur frequently among individuals with VS. This has ramifications for HIV management in Ghana, as previously suppressed individuals may potentially become infectious during the period of viral rebounds. Incidence of viral rebound decreased over time, with 43, 11, and 2 participants showing corresponding falls over 6, 12, and 18 months. As stated by Campos et al., the switch to a newer ARV like DTG in our cohort is the likely cause of the gradual decrease in viral rebounds (40).

An analysis of factors linked to viral rebounds revealed a strong correlation between lower educational attachment (up to Junior High School) and higher rebound rates. This result is consistent with that of Min et al., who found that participants with education levels equivalent to high school had a higher rebound rate than those with higher education levels (41). Our findings also show the importance of some socioeconomic determinants in HIV outcomes. Participants earning less than 500 GHS/month exhibited a marginally higher viral rebound rate than those earning ≥500 GHS/month. This trend mirrors the findings of Leddy et al., who found that low-income individuals experience worse HIV treatment outcomes (42). In this study, 70% of the participants earned less than 500 GHS/month.

These economic hardships may pose a barrier to consistent healthcare access, as limited financial resources can lead to difficulties in affording transportation to clinics or even purchasing the nutritious food necessary for optimal ART efficacy. These financial constraints could contribute to inconsistent ART adherence, thereby affecting their viral rebound rates. The predominance of female participants (68%) reflects Ghana’s HIV epidemiology, where women represent approximately two-thirds of PLWH. However, viral rebound rates did not significantly vary by gender. While our study did not explore the reasons behind this lack of disparity, other studies have found higher rebound rates in males compared to women, which is attributed to frequent ART treatment interruptions among males (43–46). Further research is required to examine this phenomenon within the Ghanaian population.

Our study reports high levels of VS over an 18-month period; however, 12% of our cohort could not achieve VS. Among the unsuppressed participants, 67% of them had low-level viremia (VL 50–999 copies/ml) and were deemed suppressed according to the WHO guidelines, often receiving little attention. However, low-level viremia is known to promote viral replication, leading to virologic failure, the emergence of drug resistance, and increased HIV transmission (47–50). It is critical to pay some attention to the unsuppressed group, including those with low-level viremia, to further reduce their VL to below-detectable levels. Regular VL testing for monitoring ART is a more sensitive, timely, and reliable method for identifying treatment failure than the clinical use of CD4^+^ T cell counts and clinical symptoms (51). Therefore, it is recommended that HIV control programs in resource-limited settings invest in infrastructure and logistics to implement regular VL measurements for all PLWH on ART.

Our data were obtained from patients enrolled in our HIV cure study, with their VLs measured regularly. In contrast, the general HIV population in Ghana does not have access to routine VL testing and is not consistently monitored for treatment failure until clinical deterioration occurs. We recommend implementing regular VL measurements as part of HIV care in Ghana to ensure effective ART outcomes. Our findings confirm viral rebound among previously suppressed participants, which could impede global efforts to end the AIDS epidemic by 2030. In our situation, this emphasizes the need to target and educate PLWH, particularly those with education up to Junior High School, about the importance of VS in controlling HIV transmission.

A major challenge in achieving sustained VS in Ghana is the sporadic nature of viral load monitoring. Strengthening routine VL testing requires addressing costs, feasibility, and potential partnerships with government and non-governmental organizations. Expanding decentralized VL testing to peripheral health centers, reducing test costs through public-private partnerships, and integrating VL monitoring into routine ART services could improve accessibility and sustainability. Furthermore, policies that promote awareness campaigns emphasizing the role of VL monitoring in HIV would support these efforts.

Our study has some limitations. First, the participants did not have regular VL measurements from the start of therapy until sampling, making it difficult to determine when they achieved VS and for how long they maintained it. Additionally, we did not assess adherence to ART in our study population. Therefore, we cannot reliably associate the observed suppression and rebound rates with adherence.

Future research should incorporate adherence assessments such as pill counts, self-reports, or biomarker tracking to better understand the impact of adherence. While sufficient for initial analysis, the sample size may limit statistical power in detecting smaller effect sizes. Future studies should consider a larger cohort to enhance the robustness of the findings. Additionally, some participants were lost to follow-up, leading to potential missing data that may introduce bias in the results. The study was conducted in three hospitals in the Greater Accra region, which may not be generalizable to PLWH in other regions of Ghana with different healthcare access. Finally, we acknowledge the possibility of recall bias arising from participants self-reporting certain variables such as age. This was mitigated by cross-verifying hospital records. However, socioeconomic variables like income could not be verified due to the lack of documented records.

## Conclusion

Despite the limitations of the study, we found high levels of VS among patients enrolled in our HIV cure study, where regular VL measurements were conducted. However, the general HIV population in Ghana lacks access to routine VL testing, which may result in different suppression levels compared to those observed in our study.

We recommend that the national HIV control program prioritize investments in infrastructure and logistics to implement regular VL measurements for all PLWH on ART. This will significantly improve ART outcomes and VS rates across Ghana’s broader HIV population. Additionally, further education on the importance of VS for PLWH with up to JHS-level education is essential to maintaining VS and achieving the third “95” of the UNAIDS 95–95-95 target by 2030.

## Data Availability

The original contributions presented in this study are included in the article and supplementary materials, further inquiries can be directed to the corresponding authors.

## Glossary

HIV: Human Immunodeficiency Virus
AIDS: Acquired Immunodeficiency Syndrome
PLWH: People living with HIV
ART: Antiretroviral therapy
SSA: Sub-Saharan Africa
NNRTI: Non-nucleoside reverse transcriptase inhibitor
NRTI: Nucleos(t)ide reverse transcriptase inhibitor
INSTI: Integrase strand transfer inhibitor
NVP: Nevirapine
EFV: Efavirenz
3TC: Lamivudine
AZT: Zidovudine
TDF: Tenofovir disoproxil fumarate
DTG: Dolutegravir
H-CRIS: HIV Cure Research Infrastructure Study
NMIMR: Noguchi Memorial Institute for Medical Research
PBMC: Peripheral blood mononuclear cells
PBS: Phosphate buffered saline
FBS: Fetal bovine serum
DMSO: Dimethyl sulfoxide
RLS: Resource-limited setting
Ml: Milliliters
μl: Microliters
